# Effect of Perioperative Dexamethasone and Different NSAIDs on Anastomotic Leak Risk: A Propensity Score Analysis

**DOI:** 10.1007/s00268-016-3620-0

**Published:** 2016-07-07

**Authors:** Christian Fredrik Rushfeldt, Uwe Conrad Agledahl, Baldur Sveinbjørnsson, Kjetil Søreide, Tom Wilsgaard

**Affiliations:** 1 Department of Gastrointestinal Surgery, Division of Surgery, Oncology and Women’s Health, University Hospital of North Norway, 9038 Tromsø, Norway; 2 Department of Surgery, Hospital of Hammerfest, 9600 Hammerfest, Norway; 3 Department of Medical Biology, Faculty of Health Sciences, UiT The Arctic University of Norway, 9037 Tromsø, Norway; 4 Department of Gastrointestinal Surgery, Stavanger University Hospital, 4068 Stavanger, Norway; 5 Gastrointestinal Translational Research Unit, Laboratory for Molecular Biology, Stavanger University Hospital, 4068 Stavanger, Norway; 6 Department of Clinical Medicine, University of Bergen, 5020 Bergen, Norway; 7 Department of Community Medicine, UiT The Arctic University of Norway, 9037 Tromsø, Norway

## Abstract

**Background:**

Perioperative use of nonsteroidal anti-inflammatory drugs (NSAIDs) is associated with risk of anastomotic leak (AL). However, concomitant use of other drugs could infer a bias in risk assessment. Thus, we aimed to interrogate the risk of AL associated with NSAIDs and steroids used perioperatively.

**Methods:**

This study includes a consecutive series of patients having surgery involving an intestinal anastomosis from Jan 2007 to Dec 2009. Data records included demographic, perioperative, and surgical characteristics; AL rates; and use of NSAIDs and steroids. Risk of leak were estimated using unadjusted and multivariable (propensity score)-adjusted logistic regression models and reported as odds ratios (ORs).

**Results:**

A total of 376 patients underwent 428 operations of which 67 (15.7 %) had AL. With no medication receivers as reference, the OR for leak when adjusted for age, sex, and propensity score was 1.07 (*p* = 0.92) for ketorolac, 1.63 (*p* = 0.31) for diclofenac and 0.41 (*p* = 0.19) for dexamethasone. Risk was increased for malignancy (OR 1.88, *p* = 0.023), use of a vasopressor (OR 2.52, *p* = 0.007), blood transfusions (OR 1.93, *p* = 0.026), and regular use of steroids (OR 7.57, *p* = 0.009).

**Conclusions:**

Other factors than perioperative drugs are crucial for risk of AL. Perioperative dexamethasone was associated with a nonsignificant reduced risk of AL.

## Introduction

Anastomotic leakage (AL) is a serious complication after intestinal surgery and is more frequent after colorectal surgery than after surgery on the small intestine [1, 2]. Several risk factors have been identified [3]. As summarized in a review by Rushfeldt et al. [4], three early clinical studies suggested that the cyclo-oxygenase-2 (COX-2)-selective NSAIDs diclofenac and celecoxib were associated with a 5-fold increase in the rate of anastomotic leaks when administered for postoperative analgesia [5–7]. These studies led to a growing interest in the possible adverse effects of NSAIDs on fresh intestinal anastomoses [8–15], and the question arose whether this was a class effect associated with the anti-inflammatory effect of NSAIDs in general, or whether the adverse effect was attributed to specific NSAIDs or subgroups of NSAIDs with a different COX selectivity [4, 16, 17]. As a consequence, if the anti-inflammatory effect per se was the reason for the anastomotic leaks, then the use of perioperative anti-inflammatory steroids should also be studied in a context with NSAID use. In this study, we have for the first time compared the effects of both NSAIDs and the commonly used steroid dexamethasone in the early postoperative phase after intestinal anastomotic surgery.

## Materials and methods

### Study population and period

The study was a consecutive series of patients operated on during a 3-year period (from Jan 1, 2007 up to and including Dec 31, 2009). Patients operated on after the study period were not eligible, as NSAIDs were abandoned for postoperative analgesia in our department due to the concerns of anastomotic leaks raised in the literature [5–7].

All patients undergoing surgery including intestinal anastomoses at the Department of Gastrointestinal Surgery, University Hospital of North-Norway were included. The study and data are reported to comply with the strengthening the reporting of observational studies in epidemiology (STROBE) recommendations for observational studies [18].

All patient data, including data on doses and duration of all medicaments given perioperatively, were consecutively stored in the electronic patient journal. The data relevant to the study were retrospectively extracted from the journal and filled into a database before undergoing statistical analysis.

### Ethics

This cohort study is categorized as a quality assurance project with no requirement for disclosure or consent according to the regulations of the Norwegian Regional Ethical Committee.

### Inclusion and exclusion criteria

We included all patients operated on with new intestinal anastomoses in the lower gastrointestinal tractus (below the ligament of Treitz), whereas an anastomosis related to an upper gastrointestinal surgery was excluded. More specifically, enteroanastomoses in roux-Y gastrectomies were excluded, whereas small bowel anastomoses after reversal of a diverting stoma, with a resection of the stoma, were included. Only a few anastomoses in the small intestine were in the jejunum (e.g., resections of Mb Crohn’s strictures), while most were in the ileum. Anastomoses with the colon, rectum, or anus are grouped as colorectal anastomoses. We excluded patients who received other NSAIDs (naproxen or ibuprofen) perioperatively, patients using NSAIDs on a routine base before the operation, and patients receiving other steroids (hydrocortisone, betamethasone, or prednisolone) perioperatively.

### Per- and postoperative use of steroids and NSAIDs

The specific dose and days of ordination of NSAIDs and steroids prescribed and received by the patient within the first 5 days after the operation were recorded, as well as steroids and NSAIDs that were already in routine use before the operation. The usual NSAIDs ordinated in the postoperative phase were either ketorolac or diclofenac. Ketorolac (30 mg) was ordinated as a single i.v. dose postoperatively on the same day as the operation, with the exception of six patients who received 15–50 mg in one or two extra doses on day 0 and one patient who received 40 mg daily during days 1–2, and the administration was defined as received or not received. Diclofenac was perorally administered as tablets from day 0, and we registered the dose of diclofenac for each day, starting with the day of operation (day 0) up to postoperative day 5 (POD 5). The use of diclofenac for this time period was defined as received or not received in the further analyses, irrespective of cumulative dose, startup day, and days of administration. Dexamethasone was the usual steroid that was ordinated per- or postoperatively, and then as single doses of 4, 8 ,16 or 24 mg. Administration of dexamethasone was defined as received or not received, irrespective of doses. Seven patients using steroids as a regular medication before the operation were included, and this was registered as a variable in the patient characteristics and adjusted for in the propensity score analysis.

### Patient and perioperative characteristics

We registered the following patient and perioperative characteristics in this study: age, sex, body mass index (BMI, kg/m^2^), preoperative albumin level (g/L), smoking, American Society of Anesthesiologists’ score (ASA score), operation for malignant or benign disease, preoperative radiotherapy, elective or emergency operation, open or laparoscopic surgery, type of anastomosis, construction of diverting stoma, use of a vasopressor, blood transfusion, and regular steroid medication. The use of a postoperative vasopressor was applied as an indirect marker for low blood pressure in the early postoperative phase. A vasopressor is defined as at least one ordination of epinephrine or norepinephrine during the first day after the operation.

### Postoperative complications

All patients’ electronic journals were searched for reoperations or readmissions for at least a year after the primary operation. AL was defined in accordance with strict definitions used by others [19, 20] as clinical leakages requiring acute surgical intervention such as a re-laparoscopy or re-laparotomy. We also included one patient with a clinical leakage and an abscess from a low colorectal anastomosis that was drained by a surgical perianal incision and perirectal tunneling, in addition to two patients operated on with low rectal resections, who did not present signs of pelvic infection before their diverting stoma was reversed after 4 and 5 months, respectively. Radiologic and clinical examinations revealed presacral abscesses and defects in the anastomoses and both ended up with rectal amputations because of hard and inflamed surrounding tissue that did not allow any repair or re-anastomosing. Although not clinically significant before reversal of the stoma, the radiological and clinical findings indicated that these two anastomotic defects must have occurred in an early phase after the primary surgery.

### Statistical analysis

The data were analyzed using the statistical software package SAS 9.4 (SAS Institute, Cary, NC). Patient and perioperative characteristics were presented as percentages and numbers for binary variables and means and standard deviations for continuous variables.

Because the use of steroids and NSAIDs were not randomly assigned in this study, potential selection biases and confounding were dealt with by developing a propensity score for the use of these medicaments. Odds ratios (ORs) for leakage were estimated using logistic regression in unadjusted; age- and sex-adjusted; and age-, sex-, and propensity score-adjusted models. A two-sided *p* < 0.05 was considered statistically significant.

## Results

A total of 376 patients who underwent 428 operations with intestinal anastomoses involving the small bowel or the colorectum were included in this study. We excluded four patients receiving perioperative NSAIDs other than ketorolac or diclofenac, eleven patients receiving perioperative steroids other than dexamethasone, one patient using NSAIDs as a regular medication, and one patient because part of the electronic journal was missing. A total of 67 operations (15.7 %) were complicated with AL, with patient, clinical, and perioperative characteristics shown in Table 1. Missing data were observed for the following variables (% missing): smoking 58 (13.6 %), albumin 31 (7.2 %), and BMI 18 (4.2 %). The crude odds ratios (OR) for leakage were significantly (*p* < 0.05) higher for benign versus malignant disease, use of a vasopressor, blood transfusion, and preoperative regular steroid medication.

**Table 1:** Patient, clinical, and perioperative characteristics for the operations (*n* = 428)

	Descr. charact. by leakage		Crude OR for leakage		
	No, *n* = 361	Yes, *n* = 67	OR	95 % CI	*p* value
Age (years)	61.8 (15.1)	60.7 (15.9)	0.96	(0.81, 1.14)	0.62
Female sex	47.9 (173)	35.8 (24)	0.61	(0.35, 1.04)	0.070
Body mass index (kg/m^2^)	25.5 (4.8)	26.2 (4.6)	1.16	(0.90, 1.50)	0.25
Albumin level (g/L)	40.9 (5.4)	41.2 (4.1)	1.06	(0.80, 1.39)	0.69
Smoking	29.1 (91)	29.8 (17)	1.04	(0.56, 1.92)	0.91
ASA (range 1–4)	2.0 (0.6)	2.1 (0.7)	1.19	(0.80, 1.76)	0.39
Malignancy versus benign disease	50.4 (182)	65.7 (44)	1.88	(1.09, 3.24)	0.023
Preop. radiotherapy	9.1 (33)	9.0 (6)	0.98	(0.39, 2.43)	0.96
Emergency versus elective surgery	11.9 (43)	11.9 (8)	1.00	(0.45, 2.24)	0.99
Laparoscopic versus open surgery	10.5 (38)	3.0 (2)	0.26	(0.06, 1.11)	0.069
Colorectal versus small bowel anast.*	73.7 (266)	85.1 (57)	2.04	(1.00, 4.15)	0.050
Diverting ileostomy	22.2 (80)	17.9 (12)	0.77	(0.39, 1.50)	0.44
Use of a vasopressor	10.3 (37)	22.4 (15)	2.52	(1.29, 4.91)	0.007
Blood transfusion	19.1 (69)	31.3 (21)	1.93	(1.08, 3.45)	0.026
Regular steroid medication	0.8 (3)	6.0 (4)	7.57	(1.65, 34.6)	0.009
Medication use					
No NSAIDs or dexamethasone	28.3 (102)	22.4 (15)	1.00	ref	
Dexamethasone only	17.5 (63)	4.5 (3)	0.32	(0.09, 1.16)	0.084
Diclofenac only	13.0 (47)	17.9 (12)	1.74	(0.75, 4.00)	0.19
Ketorolac only	6.4 (23)	6.0 (4)	1.18	(0.36, 3.90)	0.78
Combinations NSAIDs/dexam.	34.9 (126)	49.3 (33)	1.78	(0.92, 3.46)	0.088

* Anastomoses with colon, rectum, or the anus in the colorectal group

### Leak rates related to use of NSAIDs and steroids

**Fig. 1:**
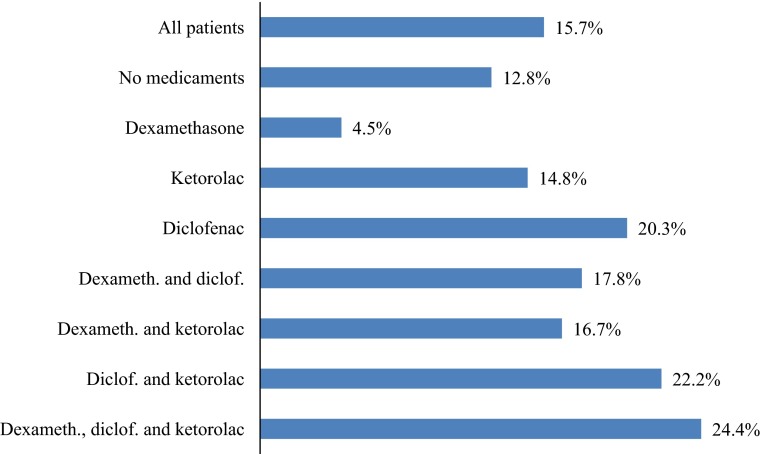
Rate of AL for different medicaments or combinations of medicaments not adjusted for other risk factors

Figure 1 illustrates the unadjusted percentage of leak rates related to the different NSAIDs or steroid used as single or combined medications. Table 2 shows the OR for leaks related to medicaments in unadjusted and multivariable-adjusted propensity score models. The perioperative use of dexamethasone as a single medication is associated with a nonsignificant decrease in AL, whereas diclofenac is associated with a nonsignificant increase in AL.

**Table 2:** Odds ratios for leaks by use of medicaments

	OR	95 % CI	*p* value
Crude data			
No use	1.00	Ref	
Dexamethasone only	0.32	(0.09, 1.16)	0.084
Diclofenac only	1.74	(0.75, 4.00)	0.19
Ketorolac only	1.18	(0.36, 3.90)	0.78
Combination use	1.78	(0.92, 3.46)	0.088
Data adjusted for age and sex			
No use	1.00	Ref	
Dexamethasone only	0.32	(0.09, 1.17)	0.085
Diclofenac only	1.74	(0.75, 4.05)	0.20
Ketorolac only	1.12	(0.34, 3.73)	0.85
Combination use	1.74	(0.87, 3.48)	0.12
Data adjusted for age, sex, and propensity score with all variables in Table 3 included			
No use	1.00	Ref	
Dexamethasone only	0.41	(0.11, 1.56)	0.19
Diclofenac only	1.63	(0.64, 4.18)	0.31
Ketorolac only	1.07	(0.31, 3.73)	0.92
Combination use	1.64	(0.75, 3.60)	0.22

*OR* odds ratio, *CI* confidence interval

### Leak rates related to type of anastomosis

Leak rates related to different type of anastomoses are shown in Table 3. Ileoileal anastomoses are complicated with a significantly lower leak rate than anastomoses involving the colon, rectum, or the anus (*p* = 0.025). Based on this finding, we distinguished between anastomoses involving the colon, rectum, or anus in one group (named “colorectal”), and those involving the small bowel only in the other group in the further analysis.

**Table 3:** Distribution of different types of anastomoses and leak rates

Type of anastomosis	No. of operations	No. of leaks	Leak rate
Small bowel	105	10	9.5 %*
Ileocolic	88	12	13.6 %
Coli-colic	39	8	20.5 %
Ileorectal	4	2	50.0 %
High colorectal**	113	21	18.6 %
Low colorectal	54	10	18.5 %
Ileoanal	16	3	18.8 %
Multiple	9	1	11.1 %
All patients	428	67	15.7 %

* *p* = 0.025

** Anastomoses defined as localized ≥7 cm above the anal verge

### Leak rates according to timing and length of administration of drugs

In Table 4, we show that the leak rate is higher if diclofenac used as a single medication was started on postoperative days 0–2 compared to days 3–5 (*p* = 0.16), and if the accumulated dose during postoperative days 0–2 was ≥250 mg (*p* = 0.22). But, since these differences were not significant, we did not discriminate between different startup days and different accumulated doses of diclofenac in the analysis of medicaments an AL. Furthermore, there were no significant differences in leak rates between the different single doses of per- or postoperative dexamethasone (Table 4). No patients in this group receiving dexamethasone as a single medication used the highest dose of 24 mg.

**Table 4:** Leak rates related to the postoperative startup day with diclofenac (POD), accumulated doses of diclofenac for days 0–2 and doses of dexamethasone given as single ordinations. Numbers relate to the groups receiving either diclofenac or dexamethasone as single medication

	Leak rate	No. of patients	No. of leaks
Startup day diclof.			
POD 0–2	24.4 %*	45	11
POD 3–5	7.1 %	14	1
Diclof. dose days 0–2			
50–200 mg	13.3 %**	15	2
250–450 mg	30.0 %	30	9
Dexamethasone dose			
4 mg	0.0 %	1	0
8 mg	2.4 %	41	1
16 mg	8.0 %	24	2

* p = 0.16

** *p* = 0.22

## Discussion

In the current study, we found a nonsignificant increase in AL with the postoperative use of diclofenac and a nonsignificant decrease in AL with a per- or postoperative use of single-dose dexamethasone when not combined with NSAIDs, after adjusting for all variables with a propensity score analysis. Other risk factors significantly associated with AL were malignancy, use of a vasopressor, blood transfusion, and use of regular steroid medication.

The initial aim of this retrospective cohort study was to investigate whether the postoperative use of NSAIDs had exerted any adverse effect on new intestinal anastomoses during a period with a high rate of AL in our department, and before we abandoned these medicaments because of the suspicion of these adverse effects. However, since it could not be ruled out that this feared complication could be a result of the NSAIDs anti-inflammatory effects acting on the healing process of the anastomosis [4], we chose to include the perioperative use of steroids, which also exert an anti-inflammatory effect. We were surprised to discover that the commonly used glucocorticoid dexamethasone given as a single dose during or shortly after an operation is associated with a nonsignificant decrease in AL when not combined with NSAIDs. Because the number of patients in the subanalysis is small and no mechanistic data exist to verify the effect, we cannot draw a direct causation related to the use of dexamethasone at this time. The findings should be considered hypothesis generating at this point, and warrant further investigation into putative mechanisms.

Dexamethasone is a potent glucocorticoid with anti-inflammatory and immunosuppressant effects, which belongs to the corticosteroids. Dexamethasone is often recommended in enhanced recovery protocols to prevent postoperative nausea and vomiting [21], to reduce postoperative pain [22, 23], and to decrease complications and length of stay after major abdominal surgery [24]. Perioperative dexamethasone does not increase infection or delay wound healing [22]. A meta-analysis of mainly randomized studies on postoperative morbidity following an esophagectomy exhibited a significant decrease in AL, as well as other serious complications after a preoperative single dose of the glucocorticoid methylprednisolone [25]. In our study, a single dose of dexamethasone administrated per- or shortly postoperatively resulted in a nonsignificant reduction of AL if the patient did not receive ketorolac or diclofenac in the early postoperative phase, thereby suggesting that these NSAIDs neutralize the protective effect of dexamethasone.

Previous studies have reported an association between the perioperative use of steroids and increased AL [26, 27]. However, the majority of these studies are based on either a high-dose and/or long-term administration of corticosteroids [28, 29] and must not be confused with single low-dose perioperative use. In our study, a perioperative single-dose therapy with dexamethasone was shown to be superior to treatment with diclofenac when comparing the risk of AL. Although both drugs are anti-inflammatory, they may exert a differential effect on the anastomotic healing process. The effect of dexamethasone on the proliferation of intestinal epithelial cells is somewhat conflicting [28–35]. Nonetheless, most studies reporting a detrimental effect on intestinal cell proliferation and tissue repair have been using relatively high concentrations of steroid drugs, as it has been shown that lower concentrations resembling those seen in a clinical setting are mostly favorable [30]. The underlying molecular mechanisms of how low-dose dexamethasone may promote tissue repair have not been revealed. Low-dose dexamethasone treatment has been reported to induce connective tissue growth factor (CTGF) in both fibroblasts and in tissues and organs after systemic administration [31, 32]. CTGF appears to play an important role in connective tissue cell proliferation and the formation of an extra cellular matrix [33–35]. A physiological role for CTGF in the wound healing process has also been emphasized [33, 36, 37], with the recent contribution of CTGF to angiogenesis revealed in experimental models [38, 39]. Thus, it seems that a single low-dose administration of perioperative dexamethasone may exert a beneficial effect on early anastomotic healing in contrast to long-term administration and/or higher doses of steroids.

In 2011, we published a review on the literature on NSAIDs and AL, and concluded in line with Klein et al. that the COX-2 selectivity of NSAIDs may be the common property that harms anastomoses [4, 19]. In this study, we recognized diclofenac as a COX-2-selective NSAID [40–42], although it is traditionally categorized as a nonselective NSAID. Other studies do not discriminate between different types of NSAIDs. In a very recent retrospective cohort study by Hakkarainen et al., 13,082 patients at 47 hospitals were categorized as either receiving or not receiving postoperative NSAIDs, independently of type/class, dose, and duration of NSAID [11]. They observed a small but significant increased risk for AL in the NSAID group for nonelective colorectal patients, thus demonstrating an unspecific class effect of NSAIDs. Bhangu et al. carried out a nonrandomized prospective multicenter study of 1503 patients in 109 centers and concluded that an early use of NSAIDs was not associated with AL [15]. However, the most frequently prescribed NSAID in this study was ibuprofen, a nonselective COX inhibitor, and in earlier studies, ibuprofen has not been associated with an increased leak rate [5, 43]. More specifically, Klein et al. evaluated the effect of NSAIDs on AL in 2766 elective patients operated on for colorectal cancer, and found that diclofenac, but not ibuprofen, was a risk factor for AL [19]. It must therefore be emphasized that the harmful effect of certain NSAIDs on anastomotic healing is not a class effect and that other NSAIDs, like ibuprofen, may be completely safe for postoperative analgesia.

With the highly COX-1-selective NSAID ketorolac [42], the results are more contradictory. In a large cohort study on 398,752 patients, a significant association (OR 1.20) between ketorolac use and AL was observed [44]. However, in a retrospective study of 731 patients undergoing colorectal surgery, no significant association between perioperative ketorolac and AL was observed [45]. This result is in accordance with the results in our study, showing almost no trend toward any increase of AL with ketorolac (OR 1.07). Thus, smaller studies may be underpowered to demonstrate a modest detrimental effect of ketorolac on AL.

In summary, the body of evidence is still in favor of the COX-2 selective NSAID diclofenac as the most counteracting NSAID for fresh anastomoses, as demonstrated in several studies [6, 7, 19, 43]. Our study with 376 patients is probably underpowered to significantly confirm this association.

The main limitation of this study is its nonrandomized, retrospective nature. However, since the primary purpose of this study was to investigate the expected detrimental effects of NSAIDs on AL, and we unexpectedly discovered a nonsignificant protective effect of dexamethasone, an observer bias is not very likely. Ketorolac and dexamethasone were administered intravenously with a low risk of false registrations. In the electronically stored medication schedules, not only the ordinations were marked, but also whether they were received or not. However, possible confounding may occur if a patient does not take an oral dose of diclofenac delivered bedside. The main results of the study were not significant, most likely indicating a somewhat undersized study sample. In order to obtain significance based on the differences observed, a 2-fold increase in the number of patients would have been needed. Thus, in the no medication group, the number of patients would have to be increased from 117 to 188, in the diclofenac group from 59 to 187, and in the dexamethasone group from 66 to 108.

A strength of this study is that the electronically stored patient data were completely except from a single patient, and that virtually all patients operated on with intestinal anastomoses during the 3-year period could be retrieved from the data system. Due to the electronic health records and the universal health insurance coverage of all patients in Norway, we are certain to have captured most likely all patients with an AL. A patient with a late presentation of AL may in theory have been managed outside our health region, but the usual practice if such an event would occur is to receive a report from the managing hospital.

The use of NSAIDs for analgesia and dexamethasone to prevent nausea and vomiting was not part of a standard analgesic or antiemetic regimen but instead depended on the personal preferences of the anesthesiologist or surgeon on duty that day, and was therefore most probably randomly administered. Even so, there might have been some degree of selection bias with regard to contraindications against steroids and NSAIDs, such as peptic ulcer disease, asthma, heart, liver, or renal failure. We did not register detailed information about comorbidity in this study, but we did adjust for ASA score, which is a physical status classification system for assessing the fitness of the patient before surgery. The severity of systemic diseases is reflected in this score, and therefore, the use of it as a variable in the propensity score analysis will reduce the selection bias with regard to medicament contraindications among patients with comorbidities [46, 47].

While ethical committees may not approve the carrying out of large scale randomized trials with diclofenac and other COX-2 selective NSAIDs with suspected dangerous effects on anastomoses and patients, a further investigation of the herein suggested protective effect of single-dose dexamethasone in randomized studies should be performed. It is important in future studies on these topics to include related medicaments, which means both NSAIDs and steroids since the perioperative use of the steroid dexamethasone may reduce the leak rate in the control groups receiving no NSAIDs.

The use of diclofenac in our department was discontinued at the end of 2009 based on the reported studies of an increased risk for AL, and the discontinuation of diclofenac was associated with a decrease in AL rate during the following years. In 2014, the use of perioperative dexamethasone was also discontinued based on an uncertainty of its role in anastomotic healing at the time. We do now plan to study the leak rate after the discontinuation of dexamethasone as well as in a prospective fashion by reinstating this medicament, and such a trial is currently being planned.

This is the first study to investigate the combined effects of both NSAIDs and the steroid dexamethasone in the perioperative setting with regard to the risk of intestinal AL. Although not significant, the results from this study generate the hypothesis that a single low dose of dexamethasone may be responsible for a reduction in AL when not combined with NSAIDs. Large, randomized multicenter studies with the administration of perioperative, single doses of dexamethasone are needed to further investigate this hypothesis.
